# Perceived loneliness, problematic pornography use, and psychological distress in Ecuadorian university students: a correlational path analysis

**DOI:** 10.3389/fpsyg.2026.1950267

**Published:** 2026-09-07

**Authors:** María Belén Morales-Cevallos, Gonzalo López-Barranco, Daniel Oleas, Jose E. Leon-Rojas, Dimitra Anastou, Jose A. Rodas

**Affiliations:** 1 Dirección de Investigación, Universidad ECOTEC, Samborondón, Ecuador; 2 Department of Social Anthropology, Basic Psychology and Public Health, Pablo de Olavide University, Dos Hermanas, Seville, Spain; 3 Department of Medicine, Universidad de Las Américas, Quito, Ecuador; 4 School of Mineral Resources Engineering, Technical University of Crete, Crete, Greece; 5 School of Psychology, University College Dublin, Dublin, Ireland; 6 Escuela de Psicología, Universidad Espíritu Santo, Samborondón, Ecuador

**Keywords:** behavioral addictions, Ecuador, loneliness, mediation analysis, problematic pornography use, psychological distress, university students

## Abstract

**Introduction:**

Loneliness is increasingly recognized as a transdiagnostic vulnerability among university students, yet the behavioral correlates associated with psychological distress remain insufficiently understood. This study examined the statistical indirect association between perceived loneliness and psychological distress via problematic pornography use among Ecuadorian university students.

**Methods:**

A quantitative, cross-sectional, correlational design was used with 273 students aged 18 to 29 years (*M* = 23.64, *SD* = 1.90) who reported habitual pornography use. Participants completed measures of perceived loneliness, problematic pornography use, and symptoms of depression, anxiety, and stress. Spearman correlations, multiple linear regression, and mediation analysis with 10,000 bootstrap resamples were conducted, controlling for age and sex.

**Results:**

Perceived loneliness was positively associated with problematic pornography use and psychological distress, and problematic pornography use was positively associated with psychological distress. In the multivariate model, loneliness and problematic pornography use were independently associated with psychological distress, whereas age and sex did not show statistically significant unique associations. The statistical indirect association between perceived loneliness and psychological distress via problematic pornography use was significant. The direct association between loneliness and psychological distress remained significant after the intermediate variable was included.

**Discussion:**

These findings suggest that problematic pornography use may represent a maladaptive behavioral correlate linking perceived social disconnection with broader emotional distress. The results also emphasize that pornography consumption should not be pathologized based on frequency alone; attention should instead focus on impaired control, functional interference, and the psychological function served by the behavior. University prevention and counseling initiatives may benefit from integrating loneliness reduction, emotion-regulation skills, social connectedness, and the non-stigmatizing assessment of problematic sexual behaviors.

## 1 Introduction

Psychological distress is a significant mental-health concern among university students. The transition to higher education frequently coincides with academic pressure, financial uncertainty, changes in interpersonal relationships, and increasing demands for autonomy (Thompson et al., 2021). These experiences may contribute to symptoms of depression, anxiety, and stress, particularly when students have limited emotional and interpersonal resources (Roy et al., 2025). In Ecuador, psychological distress is closely related to the ways individuals regulate negative emotional experiences, supporting the importance of examining both contextual vulnerabilities and coping-related processes in this population (Rodas et al., 2021, 2022; Oleas Rodríguez et al., 2024). The evidence further indicates that loneliness, perceived stress, and psychological inflexibility are associated with poorer mental health and reduced life satisfaction among university students (Yung et al., 2023; Vaca-Gallegos et al., 2025).

University students may be particularly susceptible to loneliness because emerging adulthood involves identity exploration, changes in social networks, and heightened sensitivity to interpersonal acceptance and rejection (Arnett, 2000). Loneliness should not be equated with objective social isolation (Lee and Ko, 2018), rather, it refers to the subjective perception that the quality or availability of existing relationships does not meet an individual's desired emotional or social needs (Hughes et al., 2004). Furthermore, loneliness has increasingly been conceptualized as a transdiagnostic vulnerability rather than as an isolated emotional state (Ernst, 2025). Perceived social disconnection may intensify rumination, rejection sensitivity, negative self-evaluation, and threat-related attention, while simultaneously reducing access to social support and interpersonal co-regulation (Moreta-Herrera et al., 2026). These processes may adversely affect psychological well-being and contribute directly to psychological distress. However, the association between loneliness and distress may also involve the strategies individuals use to regulate, avoid, or temporarily escape unpleasant emotional states. Even, in contemporary digital environments, such regulatory processes may also involve engagement with diverse forms of online content that provide immediate distraction, stimulation, or short-term emotional relief (Wadley et al., 2020).

Contemporary models of problematic online behavior propose that individuals may engage in immediately rewarding digital activities to obtain distraction, stimulation, or temporary relief from adverse emotional experiences (Kardefelt-Winther, 2014). Although these activities may initially reduce negative affect, repeated reliance on them can reinforce avoidance-based behavioral patterns. Over time, the behavior may become increasingly automatic or difficult to regulate, particularly when alternative coping resources are limited. This perspective does not imply that online behaviors are inherently harmful; rather, their psychological significance depends on the motives, degree of control, consequences, and functional role associated with their use (Bhatiasevi, 2024; López-Barranco et al., 2026).

Pornography use is common among adults and should not be considered pathological solely on the basis of its frequency (Bothe et al., 2020). Interpreting frequent engagement in an otherwise normative activity as evidence of addiction may contribute to the overpathologization of everyday life, whereby common behaviors are reclassified as disorders without sufficient evidence of impaired control, functional impairment, or clinically significant distress (Targhetta et al., 2013). However, some individuals report persistent difficulties controlling their pornography use, unsuccessful attempts to reduce it, continued engagement despite adverse consequences, heightened salience, and interference with everyday functioning. These characteristics are more relevant to problematic pornography use than consumption frequency alone (Bothe et al., 2024b). This distinction is particularly important because high-frequency use may occur without meaningful impairment, whereas self-perceived problems may also be shaped by moral, religious, or cultural conflict (Grubbs et al., 2019). Accordingly, problematic pornography use should be understood as a multidetermined pattern involving behavioral, affective, cognitive, interpersonal, and sociocultural factors. Moreover, pornography addiction is not recognized as an independent diagnosis in either the DSM-5-TR or the ICD-11 (Mestre-Bach et al., 2025). The ICD-11 instead includes compulsive sexual behavior disorder within the impulse-control disorders, a broader condition that may involve problematic pornography use in some cases but should not be equated with pornography addiction or frequent consumption alone (Gola et al., 2022).

Although the present study does not empirically test its specific cognitive mechanisms (e.g., cue reactivity or executive control), the Interaction of Person-Affect-Cognition-Execution (I-PACE) model provides a conceptual framework suggesting that individual predispositions interact with affective responses and executive-control difficulties to maintain problematic online behaviors (Brand et al., 2019). For individuals experiencing loneliness, pornography may serve as a highly accessible source of distraction or temporary emotional relief. Repeated reliance on this coping mechanism can reinforce expectancies regarding its regulatory effects, thereby increasing behavioral automaticity and perceived loss of control (Cardoso et al., 2022). The compensatory internet-use perspective offers a complementary account by proposing that excessive online engagement may represent an attempt to cope with adverse circumstances or unmet psychological needs rather than a purely pleasure-seeking activity (Kardefelt-Winther, 2014).

Previous findings support associations among loneliness, emotion-regulation difficulties, and problematic pornography use. Loneliness and difficulties regulating negative emotions have been independently associated with greater problematic pornography use (Cardoso et al., 2022). Indirect associations among loneliness, emotion-regulation difficulties, and problematic pornography use have also been reported, although the direction and organization of these relationships have varied across models (Cardoso et al., 2023; Vescan et al., 2024). This variability suggests that loneliness and emotion-regulation difficulties may operate within a broader network of vulnerability rather than conforming to a single universal sequence.

Problematic pornography use has also been associated with depression, anxiety, stress, loneliness, and reduced psychological wellbeing (Engelhardt et al., 2025). Nevertheless, longitudinal evidence indicates that these relationships may be more complex than a simple unidirectional causal pathway. Problematic pornography use and psychological distress appear to share relatively stable between-person associations, whereas short-term within-person effects may be smaller or inconsistent (Engelhardt et al., 2026). Additionally, pornography-use frequency alone does not adequately account for problematic use (Bothe et al., 2020).

However, the geographical concentration of previous research constitutes an additional limitation. Much of the empirical evidence on problematic pornography use has been obtained in North American, European, and selected Asian populations, though recent evidence has begun to highlight its prevalence in other specific cultural contexts (Bothe et al., 2024a; Al-Bitar et al., 2026). Cultural norms concerning sexuality, religion, family relationships, gender roles, and moral evaluations may influence pornography-use motivations, self-perceived behavioral problems, and the distress attributed to consumption. Research conducted in Ecuador has documented meaningful relationships between problematic digital behavior and psychological wellbeing among university students, suggesting that the psychological functions of online engagement warrant examination in this context (Oleas Rodríguez and López-Barranco Pardo, 2024).

Despite this growing literature, perceived loneliness, problematic pornography use, and psychological distress have often been examined as separate correlates or through direct-effect models. Consequently, it remains unclear whether problematic pornography use statistically accounts for part of the association between perceived social disconnection and broader emotional maladjustment, particularly among university students who habitually use pornography. To address this gap, the present study examined the statistical indirect association between perceived loneliness and psychological distress via problematic pornography use among Ecuadorian university students who reported habitual pornography use, while controlling for age and sex. Within the proposed model, perceived loneliness was conceptualized as an interpersonal vulnerability, problematic pornography use as a possible coping-related behavioral correlate, and psychological distress as the principal emotional outcome. Given the cross-sectional design, the model was intended to test a theoretically informed indirect association compatible with a coping-related pathway, rather than to establish causal or temporal ordering. Based on the reviewed evidence and the proposed conceptual framework, the following hypotheses were formulated:

H1. Perceived loneliness will be positively associated with psychological distress.

H2. Perceived loneliness will be positively associated with problematic pornography use.

H3. Problematic pornography use will be positively associated with psychological distress.

H4. Problematic pornography use will demonstrate a statistical indirect association between perceived loneliness and psychological distress.

## 2 Methods

### 2.1 Design

A quantitative, cross-sectional, correlational design was employed using a single administration of self-report questionnaires. The study examined the associations among perceived loneliness, problematic pornography use, and psychological distress, as well as the indirect role of problematic pornography use in the loneliness-distress association.

### 2.2 Participant

Participants were recruited through non-probability convenience sampling from several universities in Guayaquil, Ecuador. Recruitment was conducted through cross-disciplinary courses that included students from different academic programs and stages of study, a strategy that may have introduced selection bias. The sample size of 273 was deemed appropriate, as structural equation and path analyses generally require a minimum of 200 observations to achieve adequate statistical power and stable parameter estimates. Participation was voluntary, and all participants provided informed consent before completing the questionnaire. Eligibility criteria were being at least 18- years old, being currently enrolled as a university student, attending classes regularly, and reporting habitual pornography use. Habitual use was operationalized as viewing pornography on at least three separate days during a typical week.

The final sample consisted of 273 university students aged from 18 to 29 years (*M* = 23.64, SD = 1.90). Most participants were men (74.7%; *n* = 204), while women represented 25.3% (*n* = 69) of the sample. Participants were enrolled in undergraduate programs between the first and tenth academic semesters. On average, they were studying approximately the fourth semester of their degree program (*M* = 4.00, SD = 1.67).

### 2.3 Instruments

Data were collected using an online questionnaire administered through Google Forms. The questionnaire included informed consent, sociodemographic and pornography-use questions, attention-check items, and the study measures.

The first section presented the informed consent form and an *ad-hoc* sociodemographic questionnaire assessing age, sex, university, academic program, and current semester. Additional questions assessed pornography-consumption frequency and the number of days per week on which pornography was viewed. These questions were used to determine eligibility and characterize the sample.

The second section included a shortened six-item version derived from the Pornography Addiction Measurement Scale (Escala de Medición para la Adicción a la Pornografía; EMAP; Vargas Echeverría and Nevarez Martín, 2022). The six selected items (Por favor, incluyan aquí los ítems porque yo no los tengo) specifically targeted difficulty controlling use and functional interference: (1) “I watch pornography for longer than I had planned” [“Veo pornografía por más tiempo del que había planeado”]; (2) “I have tried unsuccessfully to reduce the amount of time I spend watching pornography” [“He intentado reducir el tiempo que dedico a ver pornografía sin éxito”]; (3) “I spend a lot of time thinking about pornographic content” [“Paso mucho tiempo pensando en contenido pornográfico”]; (4) “Watching pornography affects my academic or work performance” [“Ver pornografía afecta mi rendimiento académico o laboral”]; (5) “I feel irritable when I cannot watch pornography” [“Me siento irritable cuando no puedo ver pornografía”]; and (6) “I use pornography to escape from my problems” [“Uso la pornografía para escapar de mis problemas”]. Responses were recorded on a five-point Likert-type scale ranging from 1 (never) to 5 (always). A total score was calculated by summing the six items, with higher scores indicating greater problematic pornography use. In the present sample, the shortened measure showed an extremely high internal consistency (α = 0.97; ω = 0.98; mean inter-item correlation = 0.85), which may indicate a degree of item redundancy.

The third section included the three-item UCLA Loneliness Scale (UCLA-3; Russell, 1996), a brief measure of perceived loneliness. The items assess how frequently respondents feel that they lack companionship, feel left out, or feel isolated from others. In the spanish version (Pedroso-Chaparro et al., 2022) administered in the present study, items were rated on a four-point scale ranging from 1 (never) to 4 (always). A total score was obtained by summing the item responses, with higher scores indicating greater perceived loneliness. The UCLA-3 demonstrated good internal consistency in the present sample (α = 0.85; ω = 0.88 mean inter-item correlation = 0.67).

The fourth section included the 21-item Depression, Anxiety, and Stress Scale (DASS-21; Lovibond and Lovibond, 1995). The Spanish online version previously validated in Ecuadorian university students was administered (Sanmartín et al., 2022). Items assess symptoms experienced during the previous week and are rated from 0 (did not apply to me at all) to 3 (applied to me very much or most of the time). Although the DASS-21 contains three seven-item subscales, the present study used the overall score as a general indicator of psychological distress, consistent with evidence supporting a prominent common distress factor (Naumova, 2022). The total score was calculated by summing all 21 items, with higher scores indicating greater psychological distress. Internal consistency for the total score was extremely high (α = 0.99; ω = 0.99; mean inter-item correlation = 0.87), suggesting potential item redundancy or overlapping constructs within the general distress factor.

### 2.4 Procedure

Data were collected between September and October 2025 in classroom settings through an online questionnaire. The study objectives and participation conditions were explained before the informed-consent form was presented. Only students who provided electronic consent were permitted to continue. The questionnaire required completion of each section before participants could advance, preventing missing item responses.

The study adhered to the ethical principles of the Declaration of Helsinki and received approval from the Research Committee of the Psychology Program at Universidad ECOTEC (Certificate No. 19-21-09-2025). Participation was anonymous, and respondents could discontinue the survey without penalty.

### 2.5 Data analysis

Analyses were conducted in JASP (JASP Team, 2025). Internal consistency was evaluated using Cronbach's α and McDonald's ω, with ω included as a complementary reliability estimate (Trizano-Hermosilla and Alvarado, 2016). Descriptive statistics were calculated for all variables. Sex differences were examined using Welch's independent-samples t tests because of unequal group sizes. Spearman's rank-order correlations *(*ρ*)* were used to examine bivariate associations because the measures were based on ordinal response scales and some variables could depart from normality.

A multiple linear regression analysis using the enter method examined whether perceived loneliness and problematic pornography use predicted psychological distress. Age and sex were included as covariates, and all predictors were entered simultaneously to estimate their unique contributions. Independence of residuals was evaluated using the Durbin-Watson statistic. Multicollinearity was examined using tolerance and variance inflation factor (VIF) statistics. Tolerance values ranged from 0.39 to 0.97 and VIF values from 1.32 to 2.56, indicating no problematic multicollinearity.

A mediation analysis was then conducted using a regression-based path-analytic approach consistent with Hayes (Hayes, 2022). Perceived loneliness was specified as the independent variable, problematic pornography use as the mediator, and psychological distress as the dependent variable. Age and sex were entered as covariates. Indirect effects were estimated using 10,000 bootstrap resamples and percentile 95% confidence intervals (Naumova, 2022). This robust non-parametric resampling technique was explicitly selected because it does not assume normally distributed residuals, effectively accounting for potential skewness in the self-report measures and generating robust standard errors An indirect effect was considered statistically significant when its confidence interval did not include zero. Given the cross-sectional design, the mediation model was interpreted as an indirect statistical association rather than evidence of temporal or causal mediation.

## 3 Results

### 3.1 Descriptive statistics of psychological variables

Descriptive statistics and sex differences are summarized in Table 1. Women reported higher mean levels of problematic pornography use and psychological distress than men, whereas the sex difference in perceived loneliness was small and not statistically significant. Given the pronounced gender imbalance in the sample, these findings regarding sex differences must be treated as exploratory rather than confirmatory.

**Table 1:** Descriptive statistics and sex differences.

Variable	Women *M* (SD) (*n* = 69)	Men *M* (SD) (*n* = 204)	Range	*t*	*d*
Perceived loneliness	8.52 (1.56)	8.28 (2.58)	3–12	0.92	0.10
Problematic pornography use	24.82 (6.81)	18.91 (8.01)	6–30	5.95^***^	0.76
Psychological distress	49.02 (19.27)	35.19 (23.33)	0–63	4.87^***^	0.62

Cohen's d values indicate higher mean scores among women. ^***^ *p* < 001.

### 3.2 Correlations among age, perceived loneliness, problematic pornography use, and psychological distress

Spearman's rank-order correlations were calculated to examine the associations among age and the psychological variables. The results are presented in Table 2.

**Table 2:** Spearman correlations among study variables.

Variable	1	2	3
1. Age	-		
2. Perceived loneliness	−0.048	-	
3. Problematic pornography use	0.084	0.627^***^	-
4. Psychological distress	0.110	0.657^***^	0.684^***^

Values are Spearman's rank-order correlation coefficients. ^***^ *p* < 001.

### 3.3 Statistical indirect association between perceived loneliness and psychological distress via problematic pornography use

**Figure 1:**
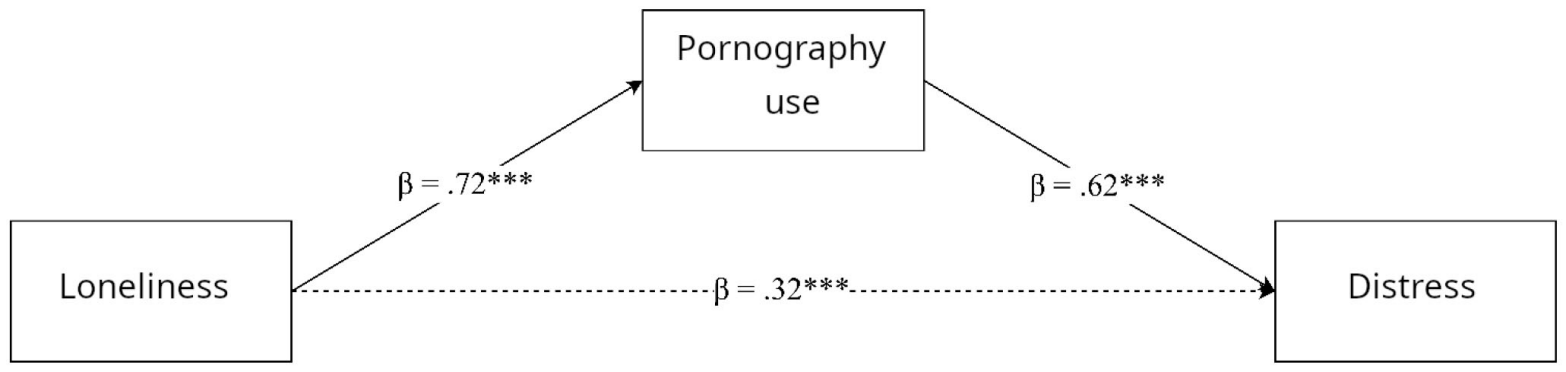
Estimated mediation model of perceived loneliness, problematic pornography use, and psychological distress. Note. Standardized coefficients are displayed in the figure. The dashed arrow represents the direct association of perceived loneliness with psychological distress after accounting for the intermediate variable. Age and sex were included as covariates in the statistical model but are omitted from the path diagram for visual parsimony. ^***^p < 0.001.

A correlational path analysis was estimated with perceived loneliness as the predictor, problematic pornography use as the intermediate variable, and psychological distress as the outcome. Age and sex were included as covariates. The model included a direct path from perceived loneliness to psychological distress, a path from perceived loneliness to problematic pornography use, and a path from problematic pornography use to psychological distress. The estimated mediation model is presented in Figure 1. Path coefficients, bootstrap confidence intervals, standard errors, standardized estimates, and direct, indirect, and total effects are reported in Table 3.

**Table 3:** Path analysis examining the association between perceived loneliness and psychological distress through problematic pornography use.

Path	*B*	95% CI	SE	β
**Direct effects on psychological distress**				
Perceived loneliness → psychological distress	3.540	[2.490, 4.629]	0.467	0.317
Problematic pornography use → psychological distress	1.708	[1.438, 1.981]	0.115	0.618
Sex (female) → psychological distress	−2.763	[−6.238, 0.744]	1.400	−0.132^a^
Age → psychological distress	0.418	[−0.141, 1.015]	0.321	0.038
**Effect on the intermediate variable**				
Perceived loneliness → problematic pornography use	2.905	[2.533, 3.310]	0.171	0.718
**Indirect effect**				
Perceived loneliness → problematic pornography use → psychological distress	4.961	[3.976, 6.000]	0.444	0.444
**Total effect**				
Perceived loneliness → psychological distress	8.501	[7.455, 9.637]	0.437	0.760

B, unstandardized coefficient; CI, confidence interval; SE, standard error; β = standardized coefficient. Confidence intervals were estimated using percentile bootstrapping. ^*a*^ Partially standardized coefficient because sex was entered as a categorical variable. Men were the reference category.

The mediation model explained 77.0% of the variance in psychological distress (*R*^2^ =0.770) and 51.5% of the variance in problematic pornography use (*R*^2^ =0.515). Neither age nor sex showed statistically significant unique associations with psychological distress in the multivariate model, as their bootstrap confidence intervals included zero.

## 4 Discussion

The present study examined the associations among perceived loneliness, problematic pornography use, and psychological distress in Ecuadorian university students who reported habitual pornography use. Overall, the findings supported the proposed hypotheses. Consistent with H1 and H2, perceived loneliness was positively associated with both problematic pornography use and psychological distress. In support of H3, problematic symptomatology maintained a substantial association with distress after age, sex, and loneliness were considered. Finally, consistent with H4, the indirect association between perceived loneliness and psychological distress through problematic pornography use was also statistically significant. However, loneliness remained directly associated with distress after the mediator was incorporated, indicating a partial statistical mediation pattern. These results suggest that the relationship between loneliness and psychological distress may involve not only the direct emotional consequences of perceived social disconnection, but also the behavioral strategies individuals use to respond to that experience. Among habitual pornography users, problematic symptomatology may constitute one coping-related process associated with this relationship. Nevertheless, because the indirect effect accounted for only part of the association, problematic pornography use should not be viewed as the sole mechanism connecting loneliness with distress.

Consistent with H1, perceived loneliness was positively associated with psychological distress, consistent with its conceptualization as a transdiagnostic vulnerability rather than simply the objective absence of social contact (Ernst, 2025). Previous research in university students has shown that loneliness is associated with poorer mental health (Hysing et al., 2026). Ecological momentary assessment research has similarly found that higher-than-usual loneliness is accompanied by immediate and day-level elevations in sadness and depression, anxiety, and stress (Kuczynski et al., 2024). Our findings extend this evidence to university students who reported habitual pornography use. During this developmental period, students must manage academic demands, changes in social networks, increasing autonomy, financial uncertainty, and decisions regarding their occupational and relational futures. When existing relationships are perceived as emotionally insufficient, these demands may become more difficult to regulate. Loneliness may intensify distress by increasing emotion-dysregulation processes while limiting access to social support and interpersonal co-regulation (Moreta-Herrera, 2024; Moreta-Herrera et al., 2026). Thus, the observed association is compatible with the view that perceived social disconnection affects emotional well-being through several interconnected cognitive, affective, and interpersonal processes.

Although female students reported higher mean levels of distress at the bivariate level, sex did not retain a statistically significant unique association with psychological distress in the multivariate regression model. This result contrasts with literature documenting a higher mental health burden among female university students (Kohls et al., 2023). A plausible explanation is that the substantial variance accounted for by perceived loneliness and problematic pornography use attenuated the unique statistical contribution of sex. Furthermore, this outcome may reflect the sampling characteristics of the study, which was restricted to students reporting habitual pornography use and was characterized by a pronounced gender imbalance (74.7% male). Consequently, the absence of a unique sex effect should be interpreted as specific to this subgroup rather than a generalizable pattern across the broader university population.

In support with H3, perceived loneliness was also positively associated with problematic pornography use. This finding is consistent with studies identifying loneliness and difficulties regulating negative emotions as correlates of greater problematic pornography use (Cardoso et al., 2022). Previous research has also documented indirect associations among loneliness, emotion-regulation difficulties, and problematic pornography use, although the direction and organization of these relationships have differed across models (Vescan et al., 2024). Rather than supporting a single universal sequence, these findings suggest that loneliness, emotional dysregulation, and problematic pornography use may form part of a broader and potentially reciprocal network of vulnerability. Pornography use may serve different functions depending on the individual's motivations, circumstances, and available coping resources. For many people, it may be related to sexual pleasure, curiosity, fantasy, or self-exploration without producing impairment. For others, however, it may provide immediate distraction from loneliness, boredom, interpersonal rejection, stress, or other unpleasant emotional states. Its accessibility, privacy, novelty, and capacity to generate rapid stimulation may make it especially reinforcing when more adaptive interpersonal or emotional-regulation resources are limited.

Consistent with H3, problematic pornography use was positively associated with psychological distress and retained a substantial unique association after perceived loneliness, age, and sex were included in the model. This result is consistent with evidence linking problematic pornography use with depression, anxiety, stress, loneliness, and poorer psychological wellbeing (Engelhardt et al., 2025). However, this relationship is more complex than a simple unidirectional causal sequence. Problematic pornography use and psychological distress appear to show relatively stable between-person associations, whereas short-term within-person effects may be smaller or inconsistent (Engelhardt et al., 2026).

Our interpretation is that problematic pornography use and psychological distress share underlying vulnerabilities and may reinforce one another over time. Distress may increase the likelihood of using pornography for relief, distraction, or emotional avoidance. When this strategy becomes repetitive, it may contribute to perceived loss of control, sleep disruption, neglect of academic responsibilities, interpersonal conflict, shame, or unsuccessful attempts to reduce consumption. These consequences may generate additional distress and increase the likelihood that pornography will again be used as a coping response. The relationship may therefore involve a reciprocal process rather than a single linear pathway. Nevertheless, this possibility cannot be established with the cross-sectional data obtained in the present study.

Consistent with H4, problematic pornography use demonstrated a statistical indirect association between perceived loneliness and psychological distress. This indirect association represents the main theoretical contribution of the study. Previous research has generally examined loneliness as a predictor of problematic pornography use (Mestre-Bach and Potenza, 2023) or psychological distress as a correlate of problematic use (Mennig et al., 2022). By integrating these variables into a single model, the present findings suggest that problematic pornography use may represent one behavioral correlate linking perceived social disconnection to broader emotional maladjustment. This indirect association may reflect an avoidance-and-reinforcement process. Students experiencing loneliness may use pornography to obtain temporary relief from emotional discomfort or to access immediate stimulation. If the behavior reduces negative affect, even briefly, its future use becomes more likely. Persistent reliance on this strategy may limit opportunities to address unmet relational needs, seek meaningful social support, or develop more flexible emotion-regulation responses. When consumption becomes difficult to control or begins to interfere with everyday functioning, it may contribute to greater distress. However, because coping motives and short-term emotional responses to pornography use were not directly assessed, this explanation should be regarded as theoretically plausible rather than empirically demonstrated by the present data.

The direct association between loneliness and psychological distress nevertheless remained significant after problematic symptomatology was included in the model. This partial mediation is theoretically plausible because psychological distress is a multidetermined outcome. Loneliness may also be associated with distress through rumination or other avoidance-based behaviors (Wang et al., 2018). Problematic pornography use should therefore be understood as one potentially relevant behavioral correlate rather than the primary or exclusive explanation of the loneliness–distress relationship.

The partial effect also prevents an overly reductionist interpretation. The findings do not indicate that loneliness is associated with distress mainly because individuals consume pornography. Instead, among students who already reported habitual use, greater problematic symptomatology statistically accounted for part of the association between perceived social disconnection and emotional distress. Given the cross-sectional design, this pattern should be interpreted as a theoretically compatible indirect association rather than evidence of temporal or causal mediation. Furthermore, it is essential to interpret the high proportion of explained variance in this model (*R*^2^ =0.770) with caution. As data on key confounders such as personality traits, relationship status, and rumination were not collected, this value could not be recalculated and is likely inflated by common-method variance, representing a methodological artifact rather than a precise estimate of predictive power.

An important conceptual limitation of the present model is the potential confounding role of moral incongruence. Because religiosity and moral beliefs regarding pornography use were not measured, it is not possible to fully disentangle functional impairment from distress arising from moral conflict. In populations where conservative religious norms are prevalent, such as Ecuador, individuals may report higher problematic use symptoms due to guilt or shame rather than actual loss of control or behavioral interference. Consequently, the interpretation of problematic pornography use in this context may overlap with general negative affectivity. Future research must incorporate measures of moral incongruence, religiosity, and specific emotion regulation strategies to isolate the functional consequences of pornography use from distress driven by moral evaluations.

## 5 Limitations

The study has several strengths. It examined a theoretically informed indirect association instead of limiting the analysis to bivariate relationships, contributed evidence from an underrepresented Latin American context, and estimated the indirect effect using 10,000 bootstrap resamples. Age and sex were also incorporated as covariates, and psychological distress was examined as a broad transdiagnostic outcome consistent with the overlap among depressive, anxious, and stress-related symptoms. Several limitations should nevertheless be acknowledged. First, the cross-sectional design precludes conclusions regarding temporal precedence and causality. Due to the cross-sectional design, all proposed mechanisms are speculative and equally compatible with reverse-causation or third-variable explanations. Alternative models, such as psychological distress predicting problematic pornography use, which in turn predicts loneliness, are highly plausible and cannot be ruled out with the current data. Second, all constructs were assessed using self-report questionnaires administered during a single session. This methodology likely inflated the observed associations —and potentially the extremely high internal consistency estimates (α ≥0.97) —through common-method variance, mood-congruent recall bias, or shared response tendencies. Additionally, the forced-response survey format prevented missing data but may have increased participant burden, potentially compromising data quality. Furthermore, given that pornography consumption is a potentially stigmatized sexual behavior, the self-reported measures are inherently susceptible to social desirability bias. The exceptionally high proportion of explained variance in psychological distress (*R*^2^ = 0.776) must therefore be interpreted cautiously as a methodological artifact of single-source data collection rather than an absolute indicator of predictive utility. Furthermore, the model is limited by the absence of unmeasured confounders, such as neuroticism or relationship status, which would likely yield more conservative estimates. Nevertheless, while the magnitude of the explained variance is inflated, this limitation does not significantly compromise the validity of the underlying structural associations observed among perceived loneliness, problematic pornography use, and psychological distress.

Third, the non-probability convenience sampling method geographically restricts the sample to Guayaquil, preventing generalization to the broader population of Ecuadorian university students. Furthermore, the findings apply only to students who report regular pornography use. Because we restricted the sample to habitual users, our findings cannot determine whether frequency moderates the association between loneliness and pornography use, and our conclusions regarding the irrelevance of frequency are constrained by this range restriction. Finally, the pronounced gender imbalance (74.7% male) may reflect both sampling bias and population-level gender differences in pornography use. Consequently, the observed sex differences are exploratory, and the disparity limits the meaningful examination of sex-specific pathways. Furthermore, the absence of data regarding specific pornography use patterns, survey completion rates, and the distribution of academic programs restricts a comprehensive sample characterization. The sample was predominantly composed of students in the early stages of their academic training (*M* = 4.00 semesters), which may limit the generalizability of these findings to advanced undergraduates.

Future research should prioritize longitudinal and ecological momentary assessment designs to clarify the temporal organization of the associations. Repeated assessments could determine whether within-person elevations in loneliness precede pornography-related urges, whether consumption provides short-term emotional relief, and whether such relief is followed by subsequent distress. Future research must test alternative and reciprocal models while explicitly measuring the mechanisms not captured in the present design. This includes assessing specific emotion-regulation strategies, relationship status, sexual orientation, and social withdrawal, to better integrate the compensatory internet use perspective. Larger and more balanced samples would additionally permit the evaluation of whether the proposed statistical associations differ across demographic and sociocultural groups.

## 6 Conclusion

Perceived loneliness was associated with psychological distress both directly and indirectly through problematic pornography use. Although problematic symptomatology accounted for a meaningful portion of this relationship, it did not fully explain it, supporting a partial statistical mediation pattern. The findings are compatible with the possibility that, among habitual pornography users, problematic use forms part of a coping-related process involving emotional avoidance and reinforcement.

At the same time, the results emphasize the importance of distinguishing pornography consumption from problematic use. Frequency alone should not be interpreted as evidence of pathology. Assessment and intervention should instead consider impaired control, functional consequences, coping motives, moral incongruence, and the interpersonal context in which pornography use occurs. Addressing loneliness, emotion-regulation resources, and social connectedness may therefore be as important as addressing the behavior itself.

## Data Availability

The raw data supporting the conclusions of this article will be made available by the authors, without undue reservation.
